# Effect of food deprivation and hormones of glucose homeostasis on the acetyl CoA carboxylase activity in mouse brain: a potential role of acc in the regulation of energy balance

**DOI:** 10.1186/1743-7075-3-15

**Published:** 2006-02-16

**Authors:** Kristophe J Karami, John Coppola, Karthik Krishnamurthy, Domingo J Llanos, Amrita Mukherjee, K V Venkatachalam

**Affiliations:** 1 College of Osteopathic Medicine, Health Professions Division, Nova Southeastern University, 3200 South University Drive, Ft. Lauderdale, Florida 33328-2018, USA; 2 Department of Biochemistry, College of Medical Sciences, Health Professions Division, Nova Southeastern University, 3200 South University Drive, Ft. Lauderdale, Florida 33328-2018, USA

## Abstract

We studied the regulation of brain acetyl CoA carboxylase (ACC) activity during food deprivation and under the influence of hormones of glucose homeostasis: glucagon and insulin. Mice were deprived of food and water for time periods of 1, 3, 6, 9, 12 and 24 hours and were then allowed to re-feed for 5, 30 and 60 minutes. Mice that were deprived for up to 6 h, and then re-fed for 60 min, consumed the same amount of food compared to the *ad libitum *(control) animals. However, after 9 h of deprivation, mice consumed only 50% of food present even after 1 h of re-feeding, compared to the controls. The ACC activity was measured in the whole mouse brain of controls and after 1, 3, 6, 9, 12, and 24 h of food deprivation. Brain extracts assayed from control mice expressed an ACC activity of 0.988 ± 0.158 fmol/min/mg tissue without citrate and 0.941 ± 0.175 fmol/min/mg tissue with citrate. After 1 h of food deprivation, the total ACC activity without citrate decreased to 0.575 ± 0.087 fmol/min/mg and in the presence of citrate, 0.703 ± 0.036 fmol/min/mg activity was measured. The citrate-dependent ACC activity decreased over time, with only 0.478 ± 0.117 fmol/min/mg of activity remaining after 24 h. Intraperitoneal (i.p.) injections of insulin, glucagon and phosphate buffered saline (PBS) were performed and whole brain ACC activity measured. After hormone administration, there were no significant differences in ACC activity in the presence of citrate. However, in the absence of citrate, there was a significant 20% decrease in ACC activity with glucagon (1.36 ± 0.09 fmol/min/mg) and a 33% increase with insulin (2.49 ± 0.11 fmol/min/mg) injections compared to PBS controls (1.67 ± 0.08 fmol/min/mg). Neuropeptide Y (NPY) levels of corresponding brain extracts were measured by ELISA (OD) using anti-NPY antibody and showed an 18% decrease upon insulin injection (0.093 ± 0.019) and a 50% increase upon glucagon injection (0.226 ± 0.084) as compared to controls injected with PBS (0.114 ± 0.040). Thus, we postulate that the changes in ACC levels under metabolic conditions would result in a fluctuation of malonyl CoA levels, and subsequent modulation of NPY levels and downstream signaling.

## Background

Acetyl CoA carboxylase (ACC) catalyzes the biotin-dependent conversion of a acetyl CoA, HCO_3_^-^, and ATP to malonyl CoA [1]. Malonyl CoA serves as an activated C-2 donor compound during fatty acid (e.g. palmitic acid) synthesis. The structure, function and regulation of ACC have been studied extensively in relation to fatty acid synthesis in liver [2-4]. There are two isoforms: ACC-1 of liver is a cytosolic isoform, which has been associated with the formation of malonyl CoA for fatty acid synthesis. The specific enzyme activity of liver ACC isolated from fasted-refed mice was approximately 0.3 units/mg. In the presence of 2 mM citrate (well above physiological concentrations) the activity was stimulated to approximately 1.5 units/mg [5]. In contrast, ACC-2 is loosely bound to the mitochondrial outer membrane and produces malonyl CoA, which inhibits carnitine acyltransferase. Thus, the malonyl CoA derived from ACC-2 serves as a regulator of fatty acid β-oxidation and energy balance [6].

**Figure 1:**
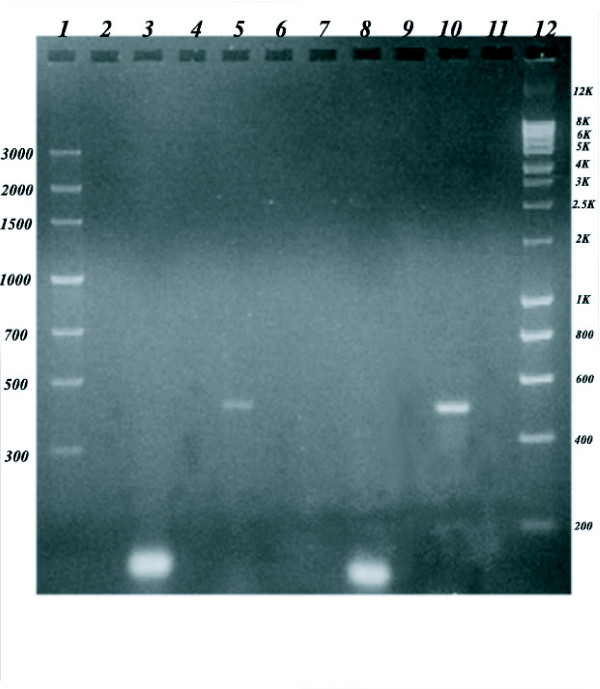
**RT-PCR of ACC-1 and ACC-2 in Mouse Brain and Liver**. ACC-2 expression levels were much higher in mouse brain as compared to ACC-1, whereas in liver, both ACC-1 and ACC-2 isoforms are expressed robustly. RT-PCR reaction was performed according to the procedures of Invitrogen, Inc. The reaction contained 0.5 μg of mouse brain or liver poly A^+ ^RNA (Clontech Inc.) and remaining components in quantities according to the manufacturer's procedure in a total volume of 50 μL. For ACC-1, the sense primer (cgaaagactcttaactctgg) and anti-sense primer (ccaggttactgatctcatct) were used. For ACC-2, the sense primer (ggaagatgacagactcgaag) and anti-sense primer (tcatcagaggagttgtcatc) were used. Lane 1: low Mw marker. Lanes 2,4,6,7,9 and 11: empty. Lanes 3 and 5: brain poly A^+ ^RNA, RT-PCR products. Lanes 8 and 10: liver poly A^+ ^RNA, RT-PCR products. The expected product sizes were confirmed referring back to the full-length cDNA.

The role of ACC in brain is poorly understood. Brain does not store fatty acids as an energy source. Brain tissue also lacks the enzymes of β-oxidation. Nonetheless, the expression of mRNA for ACC (ACC 265) isoform has been reported in developing rat brain as detected by northern blot using total RNA [7]. In this paper we report the expression of ACC-1 and ACC-2 in developing mouse brain, identified by RT-PCR using Poly A^+ ^RNA and the respective isoform specific oligonucleotides primers (Fig 1). The raison d'être of malonyl CoA synthesis in brain by ACC-1 may be explained by the existence of limited fatty acid synthesis that occurs during development and tissue regeneration. However, the expression of ACC-2 mRNA in brain is a paradox, since brain cannot use fatty acids as a fuel molecule; and therefore, has no need for carnitine acyltransferase inhibition by malonyl CoA.

Citrate serves an allosteric activator of liver ACC causing it to assume an active conformation [8]. Upon phosphorylation of ACC by various kinases, ACC activity decreases and the enzyme acquires citrate dependency for activation. Conversely, upon dephosphorylation by phosphatases, activity increases and ACC becomes less citrate-dependent. In addition to being an allosteric activator of ACC, citrate is also the source of acetyl CoA in cytoplasm for fatty acid synthesis. Therefore, citrate is also an indirect indicator of high cellular energy status [9]. The kinases and the phosphatases are under hormonal influence. Glucagon has been shown to activate the kinases and insulin activates the phosphatases [2-4].

NPY and agouti-related protein (AgRP) are orexigenic neuropeptides that regulate food intake (energy intake) and energy expenditure. Pro-opiomelanocortin (POMC) and cocaine-amphetamine-related transcript (CART) are anorexigenic neuropeptides produced by the arcuate nucleus (ARC) of the hypothalamus that also play a role in energy intake and expenditure [10]. Cerulenin (a natural product) and C-75 are inhibitors of fatty acid synthase (FAS) that cause the accumulation of malonyl CoA. In lean mice, C-75 blocked food intake and prevented fasting-induced up-regulation of hypothalamic AgRP and NPY mRNAs, as well as down-regulation of CART and POMC mRNA's [11].

In order for malonyl CoA to function as a regulator of appetite-control feeding behaviours, the presence of the ACC isoform and its regulation in brain is mandatory since malonyl CoA produced in the liver is unable to cross the blood brain barrier. We measured total ACC enzymatic activities in adult whole mouse brain after various periods of food deprivation. Here, we report the fluctuation of brain ACC activity with food deprivation and the influence of glucose homeostatic hormones: insulin and glucagon. We postulate that the malonyl CoA formed in brain by ACC may serve as a satiety sensor that would subsequently signal the production of the orexigenic (NPY) or anorexigenic (POMC) neurohormones that control feeding behaviour.

## Methods

Male BALB/c mice (retired breeders) were purchased from Charles River Labs, Inc. (Wilmington, MA). Substrates: ATP, acetyl CoA, sodium bicarbonate and hormones: insulin and glucagon, as well as anti-NPY antibody were purchased from Sigma-Aldrich, Inc. (St. Louis, MO). Radiochemicals [^14^C] H^14^CO_3_^- ^for enzyme assays were purchased from NEN Life Science Products (Boston, MA). Laboratory Rodent Diet 5001 was purchased from LabDiet. Protein detector ELISA kit, anti-rabbit ABTS systems were purchased from Kirkegaard & Perry Laboratories, Inc.

### RT-PCR

SuperScript One-Step RT-PCR with platinum Taq was used for RT-PCR. Both cDNA synthesis and PCR were performed in a single tube using gene specific primers and Poly A^+ ^RNA according to the procedures of Invitrogen, Inc. Reaction contained 0.5 μg of mouse brain or liver poly A^+^RNA and the remaining components to a final volume of 50 μL. For ACC-1, the sense primer (CGAAAGATGACAGACTCGAAG) and anti-sense primer (CCAGGTTACTGATCTCATCT) were used. For ACC-2, the sense primer (GGAAGATGACAGACTCGAAG) and anti-sense primer (TCATCAGAGGAGTTGTCATC) were used. Reverse Transcription (RT) was done for 30 min at 55°C using gene specific anti-sense primer to synthesize single stranded cDNA. PCR was performed on a Perkin-Elmer thermocycler. Thermal cycle phase consisted of 35 cycles of denaturation at 94°C for 15 s, annealing at 45°C for 30 s and extension at 68°C for 15 min. Final extension was performed for 10 min at 72°C. The PCR products were resolved on a 1% agarose gel electrophoresis using 1× TAE. The expected product sizes were confirmed referring back to the DNA sequences of the full-length cDNA.

### Starvation regimens

Mice in the experimental groups were subjected to deprivation regimens of 1, 3, 6, 9, 12 and 24 h by removing food, water and bedding. The water and the bedding were removed to emulate complete deprivation. In the control groups (*ad libitum*), food, water and bedding remained in the cages, the mice exhibiting normal feeding activity throughout the 24 h time period. The animals were initially placed one mouse/cage. To normalize diurnal variations, mice were initially exposed to 4 days of standard 12 h light/12 h dark cycles immediately upon receiving the shipment from the breeder. All groups were started with a 12 h dark cycle. Three mice were included in each group. Mice were gently asphyxiated by subjecting them to CO_2_, the whole brain carefully dissected and the tissues immediately frozen to -80°C prior to homogenization.

### Hormone injections

Mice (N = 4/group) were injected with 25 μU insulin in 0.2 mL and 2.5 μU glucagon in 0.2 mL via i.p. injection. Controls received PBS. For insulin-glucagon challenge group, glucagon (2.5 μU/0.2 mL) proceeded insulin (25 μU/0.2 mL) injection 20 min after initial injection. The insulin, glucagon and PBS groups were sacrificed after 20 min and the whole brain dissected, quickly frozen and used as needed. The insulin-glucagon challenge group was sacrificed after 40 min and the whole brain dissected and stored as in other groups. From the brain tissues, ACC activities were assayed as explained below.

### ACC activity assay

Tissues were ground in a total of 350 μL homogenization buffer [100 mM Tris-HCl (pH 8.0) containing 1 mM DTT, 1 mM EDTA, and 100 μL/100 mL of protease inhibitor cocktail (Calbiochem, Inc.)] and extracts centrifuged at 2128 × g for 5 min at 5°C. Aliquots from the supernatants were assayed using 10 μL of assay buffer [5.0 mM HEPES, 0.25 mM MnCl_2_, 0.2 mM DTT, 0.01235 mM acetyl CoA, 0.4 mM ATP, 0.05 mM citrate or replaced with water, 0.075 mg/mL BSA, 2.51 mM NaHCO_3_^- ^(containing 1.11 × 10^5 ^cpm/nmol)] added to 10 μL of tissue extracts or homogenization buffer and the reaction was incubated for 1 h at 37°C. The reaction was stopped by adding 5 μL of 1N HCl. The contents were then dried, re-suspended in 100 μL H_2_O and the radioactivity was determined using a liquid scintillation spectrometer. For each extract, ACC activity assays were performed in duplicates. The ACC activity was expressed in fmol/min/mg of brain tissue.

### Indirect antibody ELISA

Indirect antibody ELISA was performed according to the procedures of Kirkegaard & Perry Laboratories, Inc. Briefly, 2 μL of the antigens (brain extracts) were coated on to the appropriate wells by incubation overnight at 37°C. The plates were then emptied and blocked with 1× BSA for 1 h. The emptied plates were then reacted with 1:500 dilutions of rabbit anti-NPY primary antibody for 1 h at room temperature. The plates were washed three times with 1× wash solution and then reacted with secondary antibody-conjugated with alkaline phosphatase at a dilution of 1:5000 for 1 h at room temperature. The plates were washed three times with 1× wash buffer and reacted with substrate for colour development. Once the visible colour was developed, the reaction was stopped with stop solution. The plate was then read for optical density (OD) at 405 nm using Perkin Elmer Wallac 1420 VICTOR3 Multilabel Counter. For negative controls, the samples were not reacted with primary anti-NPY antibody. For blanks, the extracts were substituted with extract buffer. The net OD values for samples were obtained by subtracting the individual values with negative control values.

### Statistical analysis

For each time point, brain extracts from four animals were assayed independently. Assays were then performed in duplicates and averaged. The value in each time point represents an N of four. Enzyme activity for each was calculated and quantitative results are expressed as mean ± standard deviation (SD). Comparisons of ACC enzymatic activity groups of mouse brain homogenates were performed by one-tailed Student's *t*-test and by single factor analysis of variance (ANOVA), where appropriate.

## Results

### RT-PCR

The presence of two isoforms of ACC in mouse brain was established (Fig 1) by RT-PCR. In mouse brain, ACC-2 expression levels were much higher than levels of ACC-1 whereas, in liver, both ACC-1 and ACC-2 isoforms are expressed robustly (Fig 1). Having established the presence of ACC in mouse brain we were interested in finding the role of this enzyme in relation to food deprivation.

### Re-feeding after food deprivation

**Figure 2:**
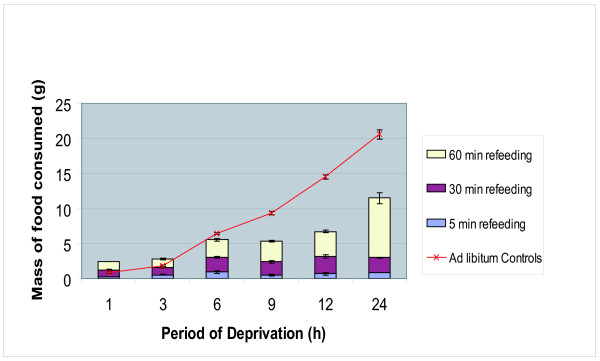
**Mass of Food Consumed during Timed Re-Feeding Periods Following Food Deprivation in the BALB/C mouse**. Food deprivation for up to 6 h, and then re-fed for 60 min consumed the same amount of food compared to the *ad libitum *(control) animals. However, after 9 h of deprivation, mice consumed only 50% of food present even after 1 h of re-feeding, compared to the controls. Retired male BALB/C breeder mice (n = 3/group) were deprived of food (LabDiet Rodent Diet 5001), water and bedding for periods of 1, 3, 6, 9, 12, and 24 h. Subsequently, re-feeding for 5, 30 and 60 min duration was carried out and the mass of food consumed during these periods was determined and compared to the mass of food consumed by the *ad libitum *controls. The analysis of variance revealed a significant difference in the *ad libitum *Control [F(5,12) = 192.67, p < 0.001], 30 min refeeding [F(5,12) = 5.87, p < 0.006] and 60 min refeeding [F(5,12) = 16.40, p < 0.001] groups. No significance was found in the 5 min refeeding group upon deprivation [F(5,12) = 1.19, p = 0.370].

We studied food consumption in adult mice after various periods of deprivation. *Ad libitum *controls consumed about 1 g of food/h continuously throughout the 24 h experimental time period. Experimental groups that were deprived of food, water and bedding for 1–6 h consumed nearly the same amount of food as controls within 1 h of re-feeding. However, after 9 h of deprivation (deprivation between 9–24 h) the mice consumed only about 50% of food compared to *ad libitum *controls even after 1 h of re-feeding (Fig 2).

### Effect of food deprivation on brain ACC activity

**Figure 3:**
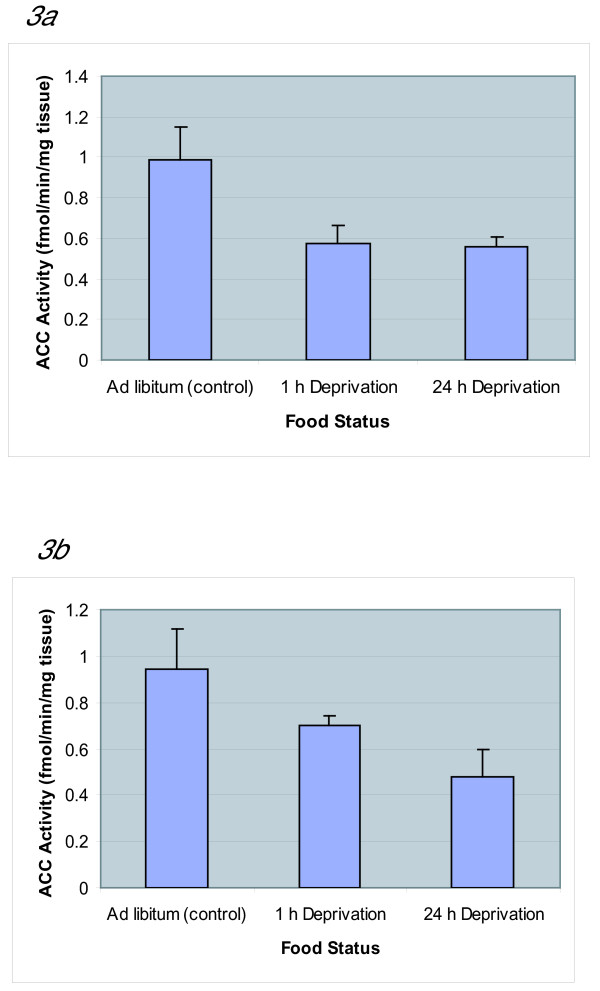
**ACC Activity in Whole Brain during Food Deprivation**. 3a (ACC, assayed without citrate). 3b (ACC, assayed with 0.05 mM citrate). The ACC activity without citrate initially decreased by 42% with no significant change between 1 h and 24 h of deprivation. In the presence of citrate, the ACC activity decreased gradually over 24 h with a significant 32% decrease at 24 h deprivation. ACC activity assayed with and without citrate of *ad libitum *(controls), 1 h deprivation, and 24 h deprivation groups are represented. Whole brain was dissected, homogenized and the extracts were assayed for ACC activity in the presence or absence of citrate. The ACC activity assay mixture contained 5.0 mM HEPES, 0.25 mM MnCl_2_, 0.2 mM DTT, 0.01235 mM acetyl CoA, 0.4 mM ATP, 0.075 mg/mL BSA, 2.51 mM NaHCO_3_^- ^(containing 1.11 × 10^5 ^cpm/nmol) as described in detail in the methods section. Results are expressed as the mean ± SD of assays of the brain extracts from four independent animals. Each extract was assayed in duplicate. A one-way analysis of variance revealed significant differences in the presence of citrate between 1 h and 24 h deprivation [F(2,6) = 9.89, p < 0.012]. When citrate was absent, significant differences between groups were also present [F(2,9) = 7.24, p < 0.013], however, no statistical significant was shown specifically between the 1 h starvation and 24 h starvation groups.

In *ad libitum *control animals, the ACC activity assayed without citrate was 0.988 (± 0.158) fmol/min/mg and was nearly unchanged in the presence of citrate (0.941 ± 0.175 fmol/min/mg) (Table 1). After 1 h of deprivation, the activity without citrate dropped significantly to 0.575 (± 0.087) fmol/min/mg and the activity with citrate was 0.703 (± 0.036) fmol/min/mg (Table 1). ACC activity without citrate remained nearly constant throughout all the deprivation regimens (1–24 h) exhibiting 0.555 (± 0.053) fmol/min/mg at 24 h deprivation (Fig 3a). However, ACC activity in the presence of citrate, from 1 h to 24 h decreased gradually to 0.478 (± 0.117) fmol/min/mg. When ACC activity with citrate at 1 h was compared with 24 h of deprivation, there is a statistically significant 32% reduction in activity (Fig 3b).

**Table 1:** ACC Activity in Whole Brain under Food Deprivation. Summary of data showing the ACC activity with and without citrate of *ad libitum *(controls) and upon 1 h, 3 h, 6 h, 9 h, 12 h, and 24 h deprivation. Each value represents an average of activities from brain extracts of four separate animals. Each extract was assayed in duplicate. Values presented as mean ± SD. A one-way analysis of variance revealed significant differences between the groups in the presence of citrate [F(6,21) = 4.47, p < 0.001] and in the absence of citrate, [F(6,21) = 3.54, p < 0.01]).

**Deprivation Regimen Time (hours)**	**ACC Activity without Citrate (fmol/min/mg tissue) ± SD**	**ACC Activity with Citrate (fmol/min/mg tissue) ± SD**
**1**	0.575 ± 0.087	0.703 ± 0.036
**3**	0.457 ± 0.117	0.607 ± 0.062
**6**	0.495 ± 0.178	0.628 ± 0.057
**9**	0.473 ± 0.138	0.597 ± 0.156
**12**	0.543 ± 0.089	0.558 ± 0.066
**24**	0.555 ± 0.053	0.478 ± 0.117
***Ad libitum *(control)**	0.988 ± 0.158	0.941 ± 0.175

### Hormonal regulation of brain ACC activity

**Figure 4:**
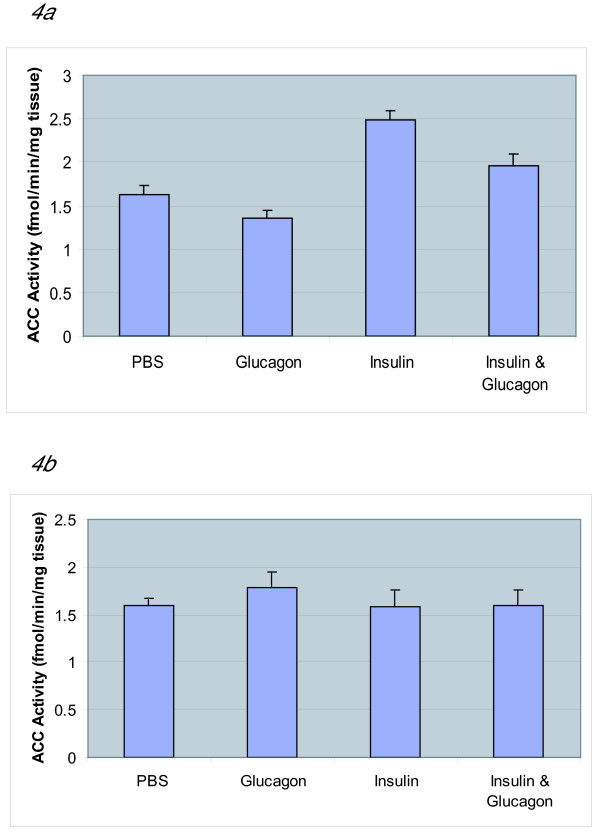
**Whole Brain ACC Activity Following Intraperitoneal Injection of PBS, Glucagon, Insulin and Insulin-Glucagon Challenge**. Figure 4a (ACC, assayed without citrate). Figure 4b (ACC assayed, with 0.05 mM citrate). ACC activity in glucagon injected mice decreased significantly in the absence of citrate and increased in the presence of citrate. ACC activity in insulin injected mice shows activity regardless of citrate presence. Retired male, BALB/C breeder mice received intra-peritoneal (i.p) injection of glucagon (2.5 μU in 0.2 mL) or insulin (25 μU in 0.2 mL). Controls received PBS. For insulin-glucagon challenge group, 20 min after insulin (25 μU/0.2 mL) injection, they received glucagon (2.5 μU/0.2 mL) injection. Whole brain was dissected, homogenized and the extracts were assayed for ACC activity in the absence of citrate. Each extract was assayed in duplicate. The ACC activity assay mixture contained, 5.0 mM HEPES, 0.25 mM MnCl_2_, 0.2 mM DTT, 0.01235 mM acetyl CoA, 0.4 mM ATP, 0.075 mg/mL BSA, 2.51 mM NaHCO_3_^- ^(containing 1.11 × 10^5 ^cpm/nmol). Results are expressed as the mean ± SD of assays of the brain extracts from four independent animals. A one-way analysis of variance revealed no significant differences in the presence of citrate between among the injection groups [F(3,12) = 1.01, p = 0.422]. When citrate was absent, significant differences between groups were found to be present [F(3,12) = 5.60, p < 0.012].

In order to understand the regulation of brain ACC activity by hormones of glucose homeostasis, we measured the ACC activity shortly after the administration of glucagon and insulin. We injected (i.p.) groups of mice with glucagon (2.5 μUnits, [12.5 ng/ml]/animal), insulin (25 μUnits, [125 ng/ml]/animal), and insulin challenged with glucagon. Control mice were injected with PBS. ACC activity assayed in the presence of citrate in the PBS-injected control group was 1.67 ± 0.08 fmol/min/mg. In the insulin-injected groups, the ACC activity was 2.49 ± 0.11 fmol/min/mg. Glucagon-injected mice exhibited ACC activity of 1.36 ± 0.09 fmol/min/mg. The insulin-glucagon challenge group exhibited ACC activity of 1.96 ± 0.14 fmol/min/mg (Fig 4a). When the ACC activity assays contained citrate the activity varied only minimally ranging from 1.56 ± 0.10 – 1.78 ± 0.16 fmol/min/mg (Fig 4b).

### Spectrophotometric Quantification of NPY

**Figure 5:**
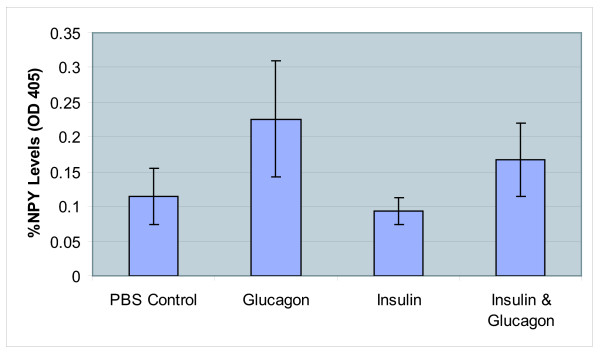
**NPY Indirect Antibody ELISA**. NPY measured in the insulin injection groups decreased 18% and in the glucagon injection groups increased 50% when compared to NPY levels of controls injected with PBS. Indirect antibody ELISA was performed according to the procedures of Kirkegaard & Perry Laboratories, Inc., using rabbit anti-NPY antibody. The NPY levels were determined through color intensity by measuring OD reading at 405 nm using a Perkin Elmer Wallac 1420 VICTOR3 Multilabel Counter. Results are expressed as the mean ± SD of assays of the brain extracts from four independent animals. Each extract was assayed in duplicate. For negative controls, the samples were not reacted with primary anti-NPY antibody. The net OD values for samples were obtained by subtracting the individual values with negative control values. The average of net OD values were converted into % change in NPY levels relative to PBS control and is plotted. A one-way analysis of variance revealed significant differences between the groups [F(3,12) = 4.13, p = 0.031].

The same extracts used for hormonal regulation of brain ACC activity measurements were utilized for the determination of NPY levels using an indirect antibody ELISA method with rabbit anti-NPY antibody. The relative values of NPY in terms of OD are presented (Fig 5). It is apparent from these results that, when compared to PBS controls (0.114 ± 0.040), extracts from insulin-administered groups possessed a modest decrease in NPY levels (0.093 ± 0.019). In contrast, NPY levels increased almost 50% in the glucagon-administered extracts (0.226 ± 0.084).

## Discussion

When mice were deprived of food, water, and bedding for up to 6 h, they retained the appetite to consume the same amount of food as controls when allowed to re-feed for 1 h. However, after 6 h of deprivation (between 9–24 h), when allowed to re-feed for up to 1 h, only 50% of the available food was consumed. This is quite interesting in that feeding behaviour appears retained for up to 6 h after deprivation (Fig 2). Thereafter, some mechanism inhibits the animals' ability to fully feed, unlike continuously fed control animals (1 g of food/h). We speculate that under periods of deprivation, mice become adapted to the lack of food and water by slowing their physiological processes in order to limit energy intake. It is interesting that this feeding behaviour in mice is manifested only after 6 h of deprivation. The biochemical basis of this process is not clearly understood.

When we performed RT-PCR using oligonucleotides that are specific to ACC-1 and ACC-2 isoforms with poly A^+ ^mRNA from developing brain, we identified the presence of both ACC-1 and ACC-2 transcripts. RT-PCR shows that the levels of ACC-1 are higher in liver than in brain while the levels of ACC-2 are nearly similar in two tissues (Fig 1). Liver is the major tissue that synthesizes fatty acids and, therefore, the role of high ACC-1 levels is clear. It is known that the cytosolic ACC-1 isoform produces the committed metabolite malonyl CoA that is channelled for *de novo *synthesis of palmitic acid. In contrast, the isoform ACC-2 that is loosely bound to the inner mitochondrial membrane has been purported to produce malonyl CoA that is involved in regulating the β-oxidation of fatty acid by inhibiting carnitine acyl transferase. Therefore, the presence of both isoforms ACC-1 and ACC-2 in liver tissue makes logical sense.

The expression of ACC-1 and ACC-2 in brain is an enigma. One might speculate that the expression of ACC-1 in brain is necessary since adult brain undergoes tissue regeneration (or neuronal regeneration) and this would imply a role of increased fatty acid synthesis. Nonetheless, the expression of ACC-2 in brain is a paradox since brain cannot use fatty acids as fuel. Therefore, an alternate explanation is warranted. We hypothesized that malonyl CoA produced by ACC-2 in brain tissues may be involved in the regulation of satiety and feeding. The role of glucose homeostatic hormones and ensuing discussion on the hormonal regulation of brain ACC was first presented by our lab [12]. Others have discussed the role of malonyl CoA in NPY regulation [13].

C-75 is a fatty acid synthase (FAS) inhibitor that causes the accumulation of malonyl CoA whereas TOFA is an ACC inhibitor, which results in the reduction of malonyl CoA synthesis. Using these inhibitors, changes in the *in vivo *levels of malonyl CoA may be elicited. It has been found that malonyl CoA concentrations correlated with mRNA expression of hypothalamic neuropeptides NPY and POMC [11]. It was also demonstrated that upon treatment with FAS inhibitors (C-75) there was reduced food intake and body weight in mice as well as increased malonyl CoA [14]. The role of malonyl CoA in relation to energy balance was clearly demonstrated by many authors in other tissues [15]. Taken together, it is reasonable to suggest that malonyl CoA may alter feeding patterns through NPY and POMC concentrations. Using rat brain, the ACC activity was measured over a period of development ranging from 0.7 months to 18 months and it was found that ACC activity decreased from 0.026 μMols/min to 0.015 μMols/min [16]. Others have also shown changes in the activities of lipogenic enzymes in the brains of developing rats during development [17]. One can assume that in *adult *brain the contribution by the ACC-1 activity would be minimal, since there is very little myelin synthesis.

Nonetheless, changes in brain ACC activity in response to deprivation of varying lengths of time, and in relation to alterations in feeding behaviour during the subsequent re-feeding has not been previously examined. The liver ACC enzyme, upon phosphorylation, becomes more citrate-dependent for catalytic activity [3]. We hypothesized that a similar mechanism may occur with the brain enzyme. In this paper, we are the first to describe the role of the allosteric effector, citrate, on brain ACC activity. Since TCA cycle is very active in brain mitochondria this metabolite would be easily available to allosterically regulate the ACC-2 bound to the inner mitochondrial membrane. It is practically impossible to distinguish the two activities of the isoforms of ACC-1 and ACC-2 in crude extracts since it would be a mixture of both isoforms. A separation of the two activities would result in a loss of quantitation. Therefore, in this paper we report the total ACC activity in whole brain under various time periods of food deprivation as well as its regulation by the glucose homeostatic hormones. We also examine the fluctuation of NPY and its correlation to metabolic status.

The observation that, in control mice, ACC activity with and without citrate remained nearly unchanged shows that under normal or non-deprived conditions ACC behaves like a dephosphorylated citrate-independent form. After 1 h of food deprivation there was an abrupt decrease (~42%) in ACC activity in the absence of citrate and over 24 h of food deprivation the ACC activity continued to drop only minimally (<5%) (Fig 3a and Table 1). In contrast, ACC activity measured in the presence of citrate showed a gradual decrease in ACC activity over 24 h of deprivation (Fig 3b and Table 1) suggesting that ACC undergoes covalent modification by phosphorylation catalyzed by cAMP kinase. Phosphorylated ACC is usually citrate dependent but it appears that, over 24 h of deprivation, ACC becomes hyper-phosphorylated, such that even in the presence of citrate its activity remains lower and is essentially citrate-independent. Based on results by Shimokawa et al. [11] the expected drop in malonyl CoA, would stimulate the expression of mRNA for the orexigenic hormone, NPY.

**Figure 6:**
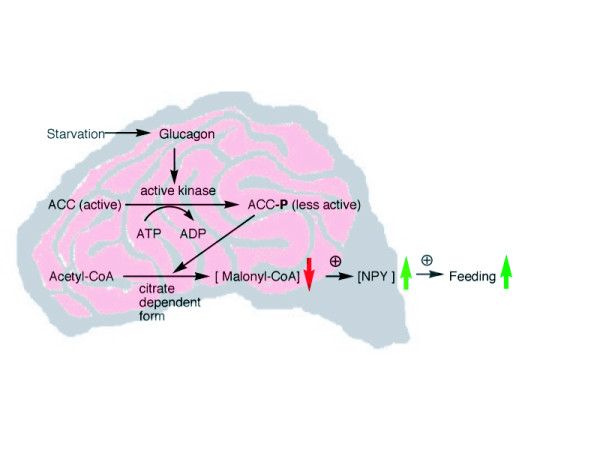
**Hypothesis of Neurohormonal Signalling During Deprivation**. Upon food deprivation, glucagon reaches a threshold level to activate the ACC-kinase. ACC-kinase phosphorylates ACC making it a less active citrate dependent species. Due to less active ACC, malonyl CoA levels drop, resulting in increased NPY expression. NPY subsequently promotes feeding.

Results from our food deprivation experiments clearly demonstrated that fluctuations in brain ACC activity correlated with metabolic status. The decrease in ACC activity with glucagon may be explained by the glucagon-induced increase in cAMP-dependent kinase activity. The kinase would be expected to phosphorylate ACC, rendering it inactive and citrate-dependent. Upon citrate binding, the phosphorylated species becomes more active and this was observed in our results (Fig 4b). Under the influence of insulin one would expect the phosphatases to be more active, re-activating ACC to its citrate-independent form. The results show that NPY levels in extracts from the glucagon injection group (0.226 ± 0.084) indicate a 50% increase in NPY when compared to PBS controls (0.114 ± 0.040) whereas the insulin injection group contained approximately 18% less NPY (0.093 ± 0.019) relative to controls (Fig 5). Thus, we postulate (Fig 6) that in the presence of glucagon, cAMP kinase phosphorylates ACC and converts it into a less active citrate-dependent species leading to decreased malonyl CoA and a subsequent rise in NPY levels. Increased NPY levels could then signal feeding behaviour. In future studies, we will be determining the NPY levels under various deprivation regimens in order to better understand the role of brain ACC with reference to the satiety and feeding.

## Conclusion

After a period of food deprivation for up to 6 h, mice were able to consume equal amounts of food within 1 h of re-feeding as compared to matched *ad libitum *controls. However, a period of deprivation from 9–24 h caused a 50% reduction in the consumption of food even after 1 h of re-feeding. ACC activity in brain fluctuates according to food deprivation. In addition, the hormones of glucose homeostasis (glucagon and insulin) may reciprocally modulate brain ACC activity. We have shown that an elevated glucagon level leads to lower ACC activity and increased NPY levels in brain. Conversely, an elevated insulin level leads to higher ACC activity and decreased NPY levels. Based on these results, we hypothesize that under periods of food deprivation glucagon levels in brain increase, stimulating cAMP dependent protein kinase. The kinase phosphorylates ACC *in vivo *converting ACC into a less active citrate-dependent form. Due to the gradual disappearance of this phosphorylated/citrate-dependent ACC species over time, there is an absolute decrease in malonyl CoA levels, correlating to the purported increase in NPY expression and the consequent promotion of feeding (Fig 6).

## Abbreviations

ACC, acetyl CoA carboxylase; FAS, fatty acid synthase; CoA, Coenzyme A; NPY, neuropeptide Y; AgRP, agouti-related protein; POMC, pro-opiomelanocortin; CART, cocaine-amphetamine-related transcript; ARC, arcuate nucleus; TCA, tricarboxylic acid; PBS, phosphate buffered saline; i.p., intra peritoneal; OD, optical density; ELISA, enzyme-linked immunosorbent assay.

## Competing interests

The author(s) declare that they have no competing interests.

## Authors' contributions

KJK: Performed most of the meticulous experimental work. Data Collection, statistical analysis, plotting and figure preparations were also performed by KJK. In addition, he discussed the work with the mentor, KVV, and also critiqued the article.

JC and KK: Performed the earlier experiments. They presented earlier works at a national student forum.

DJL: Rendered technical assistance to KJK.

KVV: The conception, design, analysis and interpretations of the experimental work. Drafting, critiquing and communication of the article is also performed by KVV.
